# A new criteria for acute on preexisting kidney dysfunction in critically ill patients

**DOI:** 10.1080/0886022X.2023.2173498

**Published:** 2023-02-02

**Authors:** Dejiang Hong, Qinghuan Ren, Jie Zhang, Fubo Dong, Shiqiang Chen, Wei Dong, Xiaoyan Chen, Longwang Chen, Yongming Yao, Zhongqiu Lu, Guangju Zhao

**Affiliations:** a Department of Emergency, Emergency Intensive Care Unit, The First Affiliated Hospital of Wenzhou Medical University, Wenzhou, PR China; b Alberta College of Wenzhou Medical University, Wenzhou, PR China; c Translational Medicine Research Center, Medical Innovation Research Division and Fourth Medical of the Chinese PLA General Hospital, Beijing, PR China

**Keywords:** Chronic kidney dysfunction, serum creatinine, mortality, acute kidney injury, critically ill patients

## Abstract

Critically ill patients with preexisting kidney dysfunction (PKD) are at high risk for acute kidney injury (AKI). Nevertheless, there is no criteria for screening and classifying AKI in patients with PKD. In this study, after assessing relationship between the change in SCr from baseline and in-hospital mortality, a new criteria, named APKD, for identifying AKI in PKD was proposed. APKD defined AKI in critically ill patients with PKD as an absolute increase of ≥ 0.2 mg/dL in SCr within 48 h or an increase in SCr ≥ 1.1 times over baseline within 7 d. APKD detected more AKI among PKD patients compared with the other criteria. Additionally, the AKI patients identified by APKD but missed by the other criteria had higher mortality than those without AKI. APKD shows higher sensitivities than KDIGO criteria in predicating in-hospital mortality. APKD, but not the KDIGO, is effective for staging the severity of AKI in patients with PKD. In conclusion, APKD is more effective in screening and classifying AKI in critically ill patients with PKD compared with the earlier criteria, and it may helpful in guiding clinical treatment and predicting prognosis.

## Introduction

Acute kidney injury (AKI), characterized by sudden decline in kidney function, is a common disease in critically ill patients [1–6]. In the past decades, many AKI diagnostic criteria have been issued to guide clinical work, such as Risk, Injury, Failure, Loss, End stage kidney disease (RIFLE) criteria, Acute Kidney Injury Network (AKIN) criteria and, more recently, Kidney Disease: Improving Global Outcomes (KDIGO) criteria [7–10]. In addition to the volume of urine output (UO), the change in serum creatinine (SCr) from baseline is recommended for the identification and staging the severity of AKI [7–10].

Baseline SCr (bSCr) used in these AKI criteria is to reflect the baseline renal function of patients before kidney injury [11–14]. Patients with bSCr within the past 12 months yielding an estimated glomerular filtration rate (eGFR) <60 mL/min/1.73 m^2^ were considered having chronic kidney disease (CKD) [15–19]. Some studies have paid attention to the particularity of CKD patients with subsequent AKI. Compared with AKI patients without CKD, patients with acute on CKD have significantly lower long-term survival rate and higher dialysis risk [13,20]. Nevertheless, the usefulness of KDIGO AKI definition and staging criteria in patients with prior kidney dysfunction (PKD) is not fully assessed.

A recent study found that the criteria based on the reference change value of SCr were more efficiency than KDIGO criteria in predicting the outcomes of AKI patients with previous CKD [21]. In intensive care unit (ICU) clinical practice, preadmission bSCr, especially the SCr levels during 7–365 d before admission, is usually unavailable [11,12]. Moreover, as a prerequisite for the diagnosis of CKD, the accurate duration of kidney dysfunction is also difficult to determine. In other words, a patient with decreased eGFR may have CKD, acute kidney dysfunction or both [4]. Thus, how to identify AKI in these patients is an important clinical issue worth of research.

In the real world, the missing values of bSCr, and relatively few patients with PKD make it difficult for clinicians to explore the above issues. The public-access ICU database based on real-world electronic medical records allows us to investigate various questions that arise in ICU clinical practice [22–24]. In this study, the critically ill subjects of Multiparameter Intelligent Monitoring in Intensive Care (MIMIC) III/IV databases and the eICU Collaborative Research Database (eICU-CRD) were enrolled to explore whether the KDIGO AKI criteria is applicable to patients with preexisting kidney dysfunction (PKD). If not, a new criterion will be designed to identify and classify acute on preexisting kidney dysfunction (APKD).

## Methods

### Data sources

This retrospective observational study was conducted using the data stored in the MIMIC-III version 1.4, MIMIC-IV version 1.0 (Boston, MA ,USA), and eICU databases version 2.0 (NY, USA). MIMIC-III and MIMIC-IV databases are large, single-center databases containing high-quality data on ICU inpatients of Beth Israel Deaconess Medical Center (BIDMC) [22,23]. eICU-CRD is a multicenter database that records the health data of critically ill patients admitted to 335 units at 208 hospitals located throughout the United States [24].

### Study population

Patients who have the data of bSCr were enrolled in this study. For patients with multiple hospitalizations, only the information of their first ICU admission was analyzed. The exclusion criteria are as follows: (1) Age <18 years; (2) ICU length of stay < 24-h; (3) History of renal transplant; (4) SCr measurement less than 2 times during ICU stay. The flow chart of included patients was shown in Supplemental Figure 1.

### Data collection

Data extraction was performed in Postgre SQL database management system using Structured Query Language (SQL). Clinical data at ICU admission, including age, gender, ethnicity, comorbidities, Simplified Acute Physiology Score II (SAPS-II) score, sequential organ failure assessment (SOFA) score, and the use of vasopressors, were recorded, respectively. eGFR was estimated based on bSCr levels and was calculated by using (CKD epidemiology collaboration) CKD-EPI creatinine equation [25–28]. All SCr measurements taken during the patient’s ICU stay, within 7-d prior to ICU stay and within the 12 months prior to hospital stay were collected. Death occurred during the hospital stay was recorded.

### Definitions

In this study, preadmission baseline SCr (pbSCr) was defined as the mean SCr during 7–365 d before admission [11,12], and the dynamic SCr within 48-h or 7-d window was referred as dynamic baseline SCr (dbSCr) [10]. We defined PKD as eGFR <60 mL/min per 1.73 m^2^ calculated based on dbSCr or pbSCr [15–19].

Three criteria were used to screen AKI in this study, including KDIGO criteria, cROCK criteria, and APKD criteria. the KDIGO criteria defined AKI as an increase in SCr by ≥0.3 mg/dL within 48 h, or an increase in SCr up to 1.5 times within the previous 7 d [10]. cROCK criteria ares a criterion which is proposed based on the reference change value of SCr. cROCK criteria defined an AKI in CKD as *a* ≥ 25% increase in SCr in 7 d [21]. APKD criteria are the criteria proposed by this study which defined AKI as absolute increase in SCr of ≥ 0.2 mg/dL within 48 h or an increase in SCr ≥1.1 times over the bSCr value within 7 d. According to KDIGO criteria, AKI Stage 2 was defined as a peak to baseline ratio of 2.0–2.9 within 7 d. Increasing in SCr to 3 times baseline, or SCr value more than 4.0 mg/dL, or receiving renal replacement treatment were classified as KDIGO Stage 3 [10]. The APKD criteria define AKI Stages 2 and 3 as SCr increases of ≥1.3 times and ≥1.9 times, respectively.

Sepsis in this study was defined as suspected or documented infection together with SOFA score ≥ 2 according to Sepsis-3 criteria [29,30].

### Statistical analysis

Among the three databases, the data of MIMIC-IV database was initially used to explore the applicability of AKI criteria in patients with or without PKD. MIMIC-III and eICU databases were used as external verification databases.

Continuous variables are presented as the means [standard deviations] or medians [interquartile range (IQR)]. Categorical variables were expressed as the number and percentage. Comparisons between groups were made by using Mann–Whitney test for continuous variables, and for categorical variables, the Chi-square test or Fishers’ exact test were performed, as appropriate.

The performance of the three AKI definitions was compared in predicting the risk of in-hospital mortality using logistic regression models. Four models were fitted to determine the potential impact of con-founders on the relationship between AKI and the outcomes. Model 1: unadjusted for any con-founders. Model 2: adjusted for age and gender. Model 3: adjusted for age, gender, and bSCr. Model 4: multivariate con-founders were adjusted, including admission age, gender, bSCr, comorbidities (myocardial infarct, congestive heart failure, liver disease, cancer, and tumor), sepsis, hypertension, vasopressors use, RRT, and SAPSII score. We determined the optimal threshold for prediction of inhospital mortality by using the Youden-Index. Based on the cutoff point, the areas under the receiver operating characteristic curve (AUCs), and the sensitivities of the models were computed and compared [31–33].

Three sets of sensitivity analyses were performed to further verify the usefulness of the three criteria in patients with PKD. First, Models 2 and 3 were used to assess the applicability of the three criteria in screening AKI and staging AKI severity. Second, the patients with sepsis-induced AKI were analyzed as an independent cohort [34–37]. Finally, in addition to dbSCr, pbSCr was used to calculate eGFR. Then, the differences between the three criteria in screening AKI and staging AKI severity were compared.

Statistical analyses were performed with the SPSS software version 26.0 (SPSS Inc., Chicago, IL) and the R software version R4.1.2 for windows. In this study, two-sided *p* value *p* < 0.05 was considered statistically significant.

## Results

### Usefulness of KDIGO AKI definition and staging criteria in patients with and without prior kidney dysfunction

After reviewing 76,540 ICU admissions in the MIMIC-IV cohort, 32,794 patients were included in this study. There were 9795 patients had PKD, and AKI was identified in 6108 (62.4%) patients: 33.4% with Stage 1, 3.8% with Stage 2, and 25.1% with Stage 3. The detailed clinical characteristics and demographics of the present cohort are presented in Table 1.

**Table 1.** Patient demographics and clinical characteristics.

Characteristics	Overall	No PKD	PKD	AKI stages according to KDIGO		
				Stage 1	Stage 2	Stage 3
Number of patients	32794	22999	9795	3269	376	2463
Admission age (median, IQR), years	68.0 [56.5, 78.6]	65.0 [53.8, 75.3]	75.2 [64.5, 84.0]	77.1 [67.1, 85.0]	78.8 [68.7, 86.0]	67.8 [56.4, 77.2]
Male (%)	18907 (57.7)	13493 (58.7)	5414 (55.3)	1838 (56.2)	169 (44.9)	1528 (62.0)
Baseline SCr (median, IQR), mg/dL	0.9 [0.6, 1.2]	0.7 [0.6, 0.9]	1.6 [1.3, 2.6]	1.5 [1.3, 1.9]	1.3 [1.1, 1.5]	3.5 [2.2, 5.2]
Peak SCr (median, IQR), mg/dL	1.1 [0.8, 1.8]	0.9 [0.8, 1.2]	2.2 [1.6, 3.9]	2.1 [1.8, 2.7]	2.9 [2.5, 3.4]	5.4 [4.3, 7.2]
eGFR (median, IQR), mL/min/1.73 m^2^	84.5 [53.3, 103.1]	96.0 [82.0, 109.6]	37.6 [21.6, 49.6]	40.6 [30.8, 50.4]	48.5 [42.6, 54.1]	15.7 [9.8, 28.3]
SAPSII (median, IQR)	36.0 [28.0, 45.0]	33.0 [26.0, 41.0]	43.0 [36.0, 53.0]	44.0 [36.0, 53.0]	49.0 [41.0, 59.0]	49.0 [40.0, 59.0]
Ethnicity (%)						
White	21957 (67.0)	15399 (67.0)	6558 (67.0)	2282 (69.8)	244 (64.9)	1406 (57.1)
Black	2957 (9.0)	1941 (8.4)	1016 (10.4)	274 (8.4)	28 (7.4)	378 (15.3)
Other	7880 (24.0)	5659 (24.6)	2221 (22.7)	713 (21.8)	104 (27.7)	679 (27.6)
Comorbidity						
Myocardial infarct (%)	6243 (19.0)	3618 (15.7)	2625 (26.8)	929 (28.4)	112 (29.8)	639 (25.9)
Congestive heart failure (%)	9675 (29.5)	4841 (21.0)	4834 (49.4)	1733 (53.0)	194 (51.6)	1149 (46.7)
Chronic pulmonary disease (%)	8229 (25.1)	5377 (23.4)	2852 (29.1)	1010 (30.9)	104 (27.7)	666 (27.0)
Liver disease (%)	4213 (12.8)	2654 (11.5)	1559 (15.9)	459 (14.0)	71 (18.9)	645 (26.2)
Diabetes (%)	7803 (23.8)	4940 (21.5)	2863 (29.2)	992 (30.3)	114 (30.3)	719 (29.2)
Renal disease (%)	6738 (20.5)	1168 (5.1)	5570 (56.9)	1884 (57.6)	162 (43.1)	1698 (68.9)
Cancer tumor (%)	5028 (15.3)	3595 (15.6)	1433 (14.6)	478 (14.6)	71 (18.9)	332 (13.5)
Sepsis (%)	14432 (44.0)	11844 (51.5)	2588 (26.4)	882 (27.0)	133 (35.4)	377 (15.3)
Hypertension (%)	18664 (56.9)	12263 (53.3)	6401 (65.3)	2198 (67.2)	279 (74.2)	1848 (75.0)
Vasopressors use (%)	13302 (40.6)	9047 (39.3)	4255 (43.4)	1476 (45.2)	212 (56.4)	1269 (51.5)
RRT (%)	2012 (6.1)	303 (1.3)	1709 (17.4)	0 (0.0)	0 (0.0)	1475 (59.9)
In hospital mortality (%)	4093 (12.5)	2109 (9.2)	1984 (20.3)	685 (21.0)	142 (37.8)	745 (30.2)

AKI: acute kidney injury; PKD: preexisting kidney dysfunction (baseline eGFR < 60 mL/min/1.73 m^2^); SCr: serum creatinine; SAPSII: simplified acute physiology score ii; RRT: renal replacement therapy.

Crude in-hospital mortality increased with the increase of the stages of AKI among patients without PKD (10.9% *vs*. 22.6% *vs.* 37.5%, *p* < 0.001). However, among patients with PKD, the mortality rates of patients with KDIGO Stage 2 was higher than those with stage 3(37.8% *vs.* 30.2%, *p* = 0.003) (Supplemental Figure 2). The results of logistic regression analysis showed that PKD patients with AKI Stage 2 presented higher OR compared with those with AKI Stage 3 (Unadjusted OR, 4.82; 95% CI, 3.82–6.08 *vs*. OR, 3.45; 95% CI, 3.02–3.94) (Supplemental Figure 3). The results were validated in MIMIC-III and e-ICU cohorts (Supplemental Figure 4).

### A new criterion for the definition and classification of APKD

**Figure 1.**
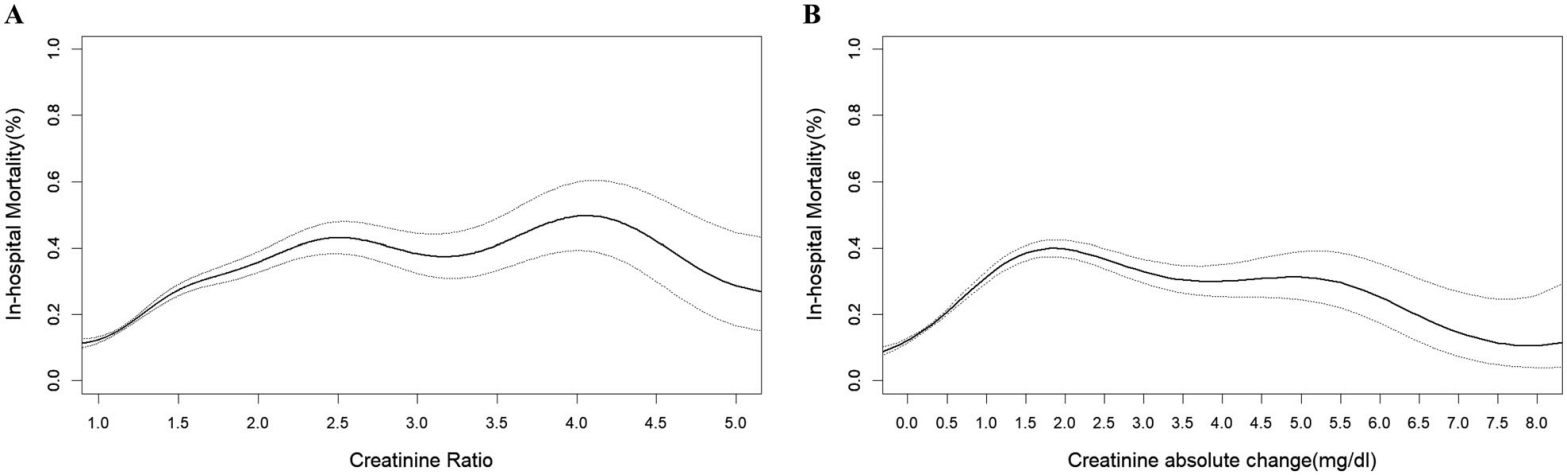
Association of the ratio of pSCr/bSCr and the absolute increase in SCr (pSCr-bSCr) with in-hospital mortality in patients with PKD. PKD: preexisting kidney dysfunction. pSCr: peak serum creatinine; bSCr: baseline serum creatinine.

Generalized additive models showed that the SCr ratio (pSCr/bSCr) and the absolute rising values of SCr (pSCr-bSCr) had nonlinear and asymmetric relationships with mortality (Figure 1(A,B)). Then, patients were divided into 16 groups and 21 groups on the basis of the SCr ratio and the absolute change values of SCr, respectively.

As shown in Supplemental Figure 5, in MIMIC-IV cohorts, the hospital mortality of patients with absolute increase in SCr of 0.1–0.19 mg/dL was comparable to those with 0.0–0.1 mg/dL (9.1% *vs.* 11.2%, *p* = 0.151), but was lower than those with absolute change in SCr ≥ 0.2 mg/dL (11.2% *vs.* 17.0%, *p* < 0.001). Additionally, patients with the SCr ratio of 1.1–1.19 had higher hospital mortality compared those with the SCr ratio of 1.0–1.09 (11.9% *vs.* 15.9%, *p* < 0.001), but the mortality was similar between 1.1–1.19 group and 1.2–1.29 group (15.9% *vs.* 17.5%, *p* = 0.213). There is a significant difference in mortality between 1.2–1.29 group and 1.3–1.39 group (17.5% *vs.* 21.9%, *p* = 0.012). According to these results, an absolute increase in SCr of ≥ 0.2 mg/dL within 48 h or an increase in SCr ≥1.1 times over baseline within 7 d was considered for the diagnosis of AKI among patients with PKD. An increase in SCr ≥1.3 times and ≥1.9 times baseline were defined as APKD Stages 2 and 3, respectively.

### Comparison of the incidence and outcomes of AKI defined by different criteria among patients with PKD

In MIMIC-IV cohort, the incidence of AKI in patients with PKD based on KDIGO, cROCK, and APKD were 62.4%, 46.3%, and 78.3%, respectively. Compared with KDIGO and cROCK criteria, APKD identified 1563 (15.9%) and 3138 (32.0%) more AKI patients. In MIMIC-III and e-ICU cohort, APKD also detected more AKI among PKD patients compared with KDIGO or cROCK.

The crude in-hospital mortality of AKI patients diagnosed based on the three criteria was significantly higher than that of non-AKI patients (all *p* < 0.001). Nevertheless, as shown in Table 2, the patients identified by AKPD but missed by KDIGO or cROCK had higher mortality than those with no AKI based on APKD in MIMIC-III/IV and e-ICU cohorts (all *p* < 0.01).

**Table 2.** Comparison of the incidence and outcomes of AKI defined by different criteria among patients with PKD.

Definition	NOAKI (*n*%)	AKI (*n*%)	*p* Value	NOAKI (mortality, *n* (%))	AKI (mortality, *n* (%))	*p* Value
MIMIC-IV						
KDIGO	3687 (37.6)	6108 (62.4)	<0.001	412 (11.2)	1572 (25.7)	<0.001
cROCK	5262 (53.7)	4533 (46.3)	<0.001	711 (13.5)	1273 (28.1)	<0.001
APKD	2124 (21.7)	7671 (78.3)	<0.001	213 (10.0)	1771 (23.1)	<0.001
MIMIC-III						
KDIGO	2916 (37.9)	4774 (62.1)	<0.001	417 (14.3)	1217 (25.5)	<0.001
cROCK	4053 (52.7)	3637 (47.3)	<0.001	687 (17.0)	947 (26.0)	<0.001
APKD	1375 (17.9)	6315 (88.1)		176 (12.8)	1458 (23.1)	
eICU						
KDIGO	9257 (39.1)	14411 (60.9)	<0.001	1187 (12.8)	3471 (24.1)	<0.001
cROCK	13608 (57.5)	10060 (42.5)	<0.001	2050 (15.1)	2608 (25.9)	<0.001
APKD	5361 (26.7)	18307 (77.3)	<0.001	646 (12.1)	4012 (21.9)	<0.001

Definition	NOAKI APKD (mortality, *n* (%))	AKI missed (mortality, *n* (%))	*p* Value	NOAKI APKD	AKI missed HR (95% CI)	*p* Value
MIMIC-IV						
KDIGO	213 (10.0)	199 (12.7)	0.010	Reference	1.31 ( 1.07 − 1.61 )	0.010
cROCK	213 (10.0)	498 (15.9)	<0.001	Reference	1.69 ( 1.43 − 2.01 )	<0.001
MIMIC-III						
KDIGO	176 (12.8)	241 (15.6)	0.029	Reference	1.26 ( 1.02 − 1.56 )	0.029
cROCK	176 (12.8)	511 (19.1)	<0.001	Reference	1.61 ( 1.34 − 1.94 )	<0.001
eICU						
KDIGO	646 (12.0)	541 (13.9)	0.009	Reference	1.18 ( 1.04 − 1.33 )	0.009
cROCK	646 (12.0)	1404 (17.0)	<0.001	Reference	1.50 ( 1.36 − 1.66 )	<0.001

AKI: acute kidney injury; PKD: preexisting kidney dysfunction (baseline eGFR < 60 mL/min/1.73 m^2^); SCr: serum creatinine; APKD: acute on preexisting kidney dysfunction.

### Comparing the performance of in-hospital mortality predictive models between APKD and KDIGO staging criteria

As shown in Table 3, AKI defined by APKD and KDIGO criteria showed similar risk of death (OR, 2.40; 95% CI, 2.05–2.81 and OR, 2.43; 95% CI, 2.15–2.75). Apart from this, no significant differences in the AUC between the APKD and KDIGO predictive models were observed in MIMICIV cohorts (AUC = 0.730 *vs.* 0.731, *p* = 0.868). Nevertheless, using the maximum value of Youden index (Sensitivity + Specificity − 1) as a criterion for selecting the optimum cutoff value. APKD staging criteria show higher sensitivities than KDIGO criteria in predicating in-hospital mortality (0.669 *vs*. 0.638, *p* < 0.001).

**Table 3.** Performance of in-hospital mortality predictive models between APKD and KDIGO in different databases.

Definition	OR	95% CI of OR	AUC	*p* Value difference in AUC	Sensitivity, %	95% CI for Sensitivity, %	*p* Value difference in sensitivity
MIMIC-IV							
APKD	2.40	2.05 − 2.81	0.730	Reference	0.669	0.649 − 0.690	Reference
KDIGO	2.43	2.15 − 2.75	0.731	0.868	0.638	0.616 − 0.659	<0.001
MIMIC-III							
APKD	1.91	1.61 − 2.28	0.686	Reference	0.665	0.642–0.688	Reference
KDIGO	1.87	1.64 − 2.13	0.689	0.354	0.594	0.569–0.618	<0.001
eICU							
APKD	1.96	1.79 − 2.15	0.697	Reference	0.670	0.656 − 0.683	Reference
KDIGO	2.16	1.93 − 2.24	0.696	0.885	0.629	0.615 − 0.643	<0.001

AKI: acute kidney injury; PKD: preexisting kidney dysfunction (baseline eGFR < 60 mL/min/1.73 m^2^); SCr: serum creatinine; APKD: acute on preexisting kidney dysfunction; OR: odd ratio; AUC: area under curve; CI: confidence interval.

### APKD criteria in staging the severity of AKI in patients with prior kidney dysfunction

**Figure 2.**
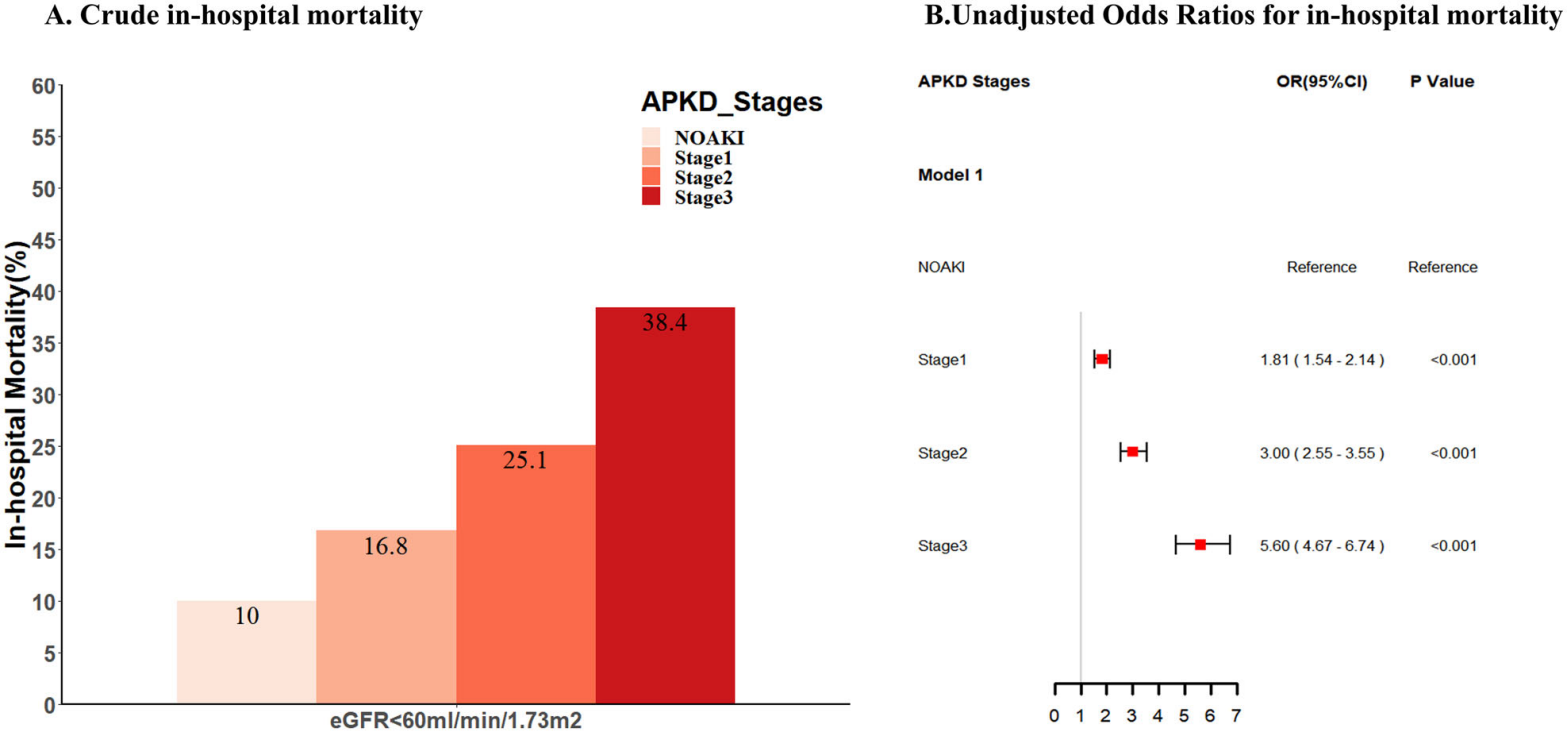
The crude and Logistic regression analyses of in-hospital mortality by APKD AKI severity stage in patients with PKD. AKI: acute kidney injury; PKD: preexisting kidney dysfunction (baseline eGFR < 60 mL/min/1.73 m^2^).

According to APKD criteria, there were 3737(38.1%) patients with AKI Stage 1, 2750 (20.1%) with Stage 2 and 1184 (12.1%) with Stage 3. The mortality of no-APKD and APKD stage 1–3 were 10.0%, 16.8%, 25.1%, and 38.4%, respectively. Crude in-hospital mortality of AKI patients diagnosed based on APKD criteria was higher than that with non-AKI, and it increased with the increase of the stages of APKD (Figure 2(A)). Using the logistic regression model, the risk of death was also increased with the increase of the stages of APKD (OR [95% CI]; 1.81 [1.54–2.14], OR [95% CI]; 3.00 [2.55–3.55], and OR [95% CI]; 5.60 [4.67–6.74]) (Figure 2(B)). The applicability of the APKD criteria in staging the severity of APKD patients was also confirmed in MIMIC-III and e-ICU cohorts (Supplemental Figures 6 and 7).

### Sensitivity analysis

Three sets of sensitivity analyses were performed to evaluate the usefulness of APKD criteria.

First, three multivariate logistic regression models (Models 2–4) were used to analysis, the associations between AKI stages according to APKD and KDIGO and in-hospital mortality. Similar results were obtained from the unadjusted and adjusted models (Supplemental Figure 8).

Sepsis is one of the main causes of AKI among critically illness patients [34–37]. Compared with KDIGO and cROCK, APKD detected more AKI in septic patients. The results also showed that, the crude mortality and unadjusted and adjusted ORs for death were similar between KDIGO AKI Stages 2 and 3 among patients with PKD (Supplemental Figures S9(A) and S10(A)). Nevertheless, the APKD staging criteria applied to this study population showed a good separation between non-AKI and different stages of AKI (Supplemental Figure S11).

Finally, the results were validated when eGFR were calculated based on the values of pbSCr. When using pbSCr-based eGFR, there were 2549 (44.6%), 2241 (39.2%), and 3142 (54.9%) AKI subjects met KDIGO, cROCK, and APKD criteria, respectively. APKD AKI staging criteria, but not the KDIGO staging criteria, is effective for staging the severity of patients with eGFR less than 60 mL/min/1.73 m^2^ (Supplemental Figure S9(B) and Figure S10(B), and Figure S12).

## Discussion

Accurate identification of AKI and staging of its severity are crucial for guiding clinical treatment and predicting prognosis. According to KDIGO criteria, the severity of AKI was classified according to the increased levels of SCr and decreased urinary volume [10]. In many studies, including randomized controlled trials, SCr-based criteria were used to enroll patients with AKI and classify their severity [20,38,39]. Nevertheless, the efficiency of SCr-based criteria in screening AKI may be influenced by the baseline kidney function. In this study, a new criterion was proposed to define AKI in patients with prior kidney dysfunction (APKD criteria). The results showed that APKD could detect about 15% more AKI events in patients with PKD than KDIGO. According to cROCK, criteria for acute on CKD, more than one half of patients with PKD are defined as non-AKI, and APKD could identify about 30% more AKI events than it. Moreover, the mortality of patients with AKI identified by APKD, but not KDIGO and cROCK, was significantly higher than non-AKI patients.

The levels of bSCr or reference SCr vary from patient to patient. Thus, instead of the peak of SCr, increasing SCr from baseline is used as the marker to diagnosis AKI in all AKI criteria [7–10]. Nevertheless, the normal variability of it should also be considered. The reference change value of SCr is within the range of 14–25% in healthy adults, and it was estimated at 20–30% in patients with eGFR< 60 mL/min/1.73 m^2^ [21]. Accordingly, a criterion based on the reference change value of SCr was proposed to define AKI in patients with CKD [21]. However, only using the distribution of reference change value to determine the cutoff value of it for the diagnosis of AKI maybe not reasonable for critically ill patients, because they are at high risk for AKI. For them, the normal variability of reference change value estimated by the distribution of it may be not ‘normal’. In this study, we analyzed the relationship between reference change value and in-hospital mortality, and the differences in mortality between different ranges of reference change value were carefully compared. The results showed that an absolute change values ≥ 0.2 mg/dL within 48 h or an increase in SCr ≥1.1 times over baseline within 7 d are associated with increased mortality in critically ill patients. Interestingly, a recent study found that a very small variation of SCr (>0.05 mg/dL) was associated with increased in-hospital mortality in the novel coronavirus disease 2019 (COVID-19) patients [40].

Currently, the variability of bSCr definition in different studies may lead to heterogeneity in reported AKI frequency and prognosis. dbSCr and pbSCr are widely used as reference SCr for diagnosing and staging AKI [11,20,39,41]. While both dbSCr and pbSCr are unavailable, the equations, including CKD-EPI and MDRD, are recommended to estimating the value of bSCr (estimated bSCr) [8,42–45]. Nevertheless, this back-estimation method defaults that the basic renal function of the patient is normal, which undoubtedly leads to an erroneous estimation of the bSCr of some patients [25,27,28,46]. Thus, only patients with actual measured SCr values were included in this study. We found that, although the values of dbSCr and pbSCr are different in some patients (data not shown), APKD criteria is effective in screening and classifying AKI in patients with PKD. Moreover, the usefulness of APKD criteria was also validated in different populations and in different databases.

There are some limitations of this study. First, as an observational study using the data of electronic databases, the results might be affected by residual confounding factors that are not adjusted or cannot be adjusted. Second, UO-based criteria are not used for diagnosing AKI in this study. Some studies found that diuresis is a more sensitive marker of AKI than SCr [47–49]. Nevertheless, in the present cohort, using KDIGO UO criteria, there is no significantly difference in in-hospital mortality between PKD patients with AKI Stage 1 and those without AKI (10.2% *vs.* 8%, *p* = 0.058). The mortality of the patients with AKI Stages 1 and 2 is also similar, although there is a statistical difference (10.2% *vs.* 13.4%, *p* = 0.024). Therefore, how to identify AKI in patients with PKD using UO-based criteria deserves further study. Thirdly, although in-hospital mortality was recorded as the endpoint of AKI, economic and long-term mortality and quality-of-life of the patients were not recorded. Finally, the APKD criteria need to be further validated in non-ICU patients.

## Conclusions

APKD is more effective in screening and classifying AKI in critically ill patients with PKD compared with earlier criteria. The results need to be verified in the future by multicenter randomized controlled trials.

## Ethical approval

MIMIC III/IV database used in this study was approved by the Institutional Review Boards (IRB) of the Massachusetts Institute of Technology and does not contain protected health information. Details of the ethical activities of the eICU database are available on the official website (https://eicu-crd.mit.edu).

## Supplementary Material

Supplemental Material

## Data Availability

Source data for information presented in this study are available from the corresponding authors on reasonable request.
