# Supplementary material for: A new criteria for acute on preexisting kidney dysfunction in critically ill patients
Source: Ren Fail. 2023 Feb 2;45(1):2173498. doi: 10.1080/0886022X.2023.2173498 (PMC9897760; doi:10.1080/0886022X.2023.2173498)
Supplement: Supplemental Material [file IRNF_A_2173498_SM6721.pdf]

Supplemental material

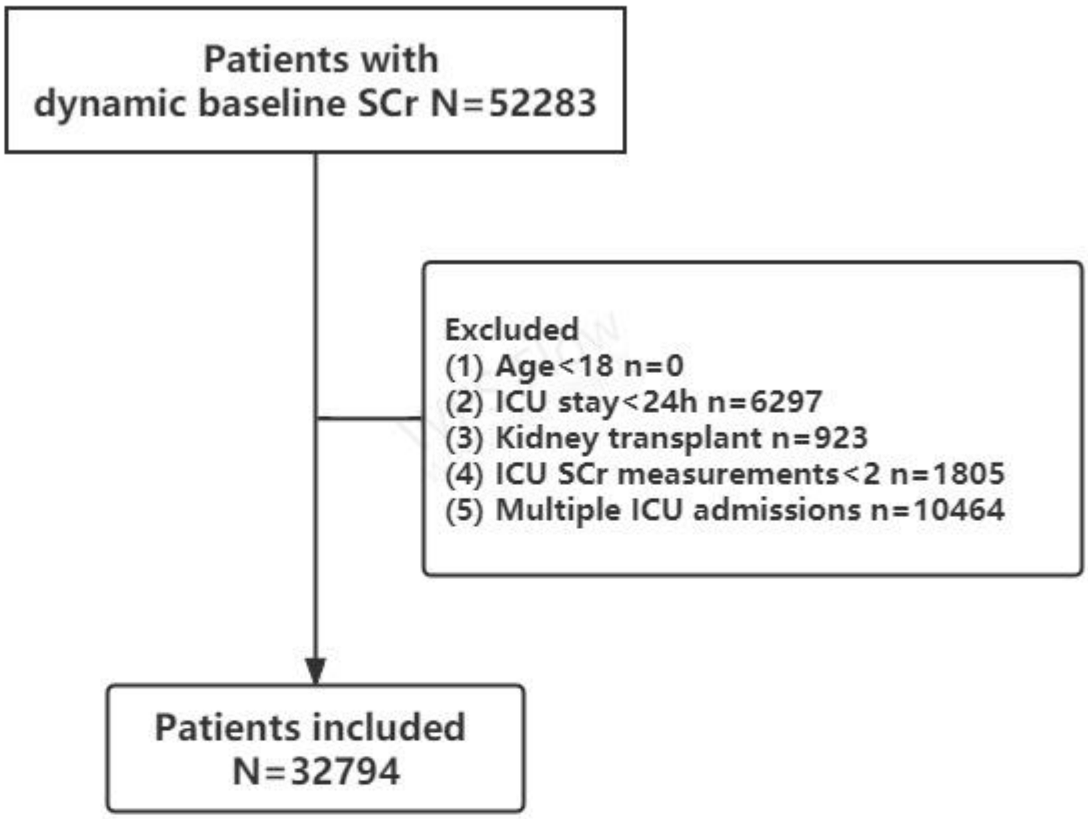

Figure S1. Flowchart of included patients. ICU, intensive care unit; SCr, serum creatinine.

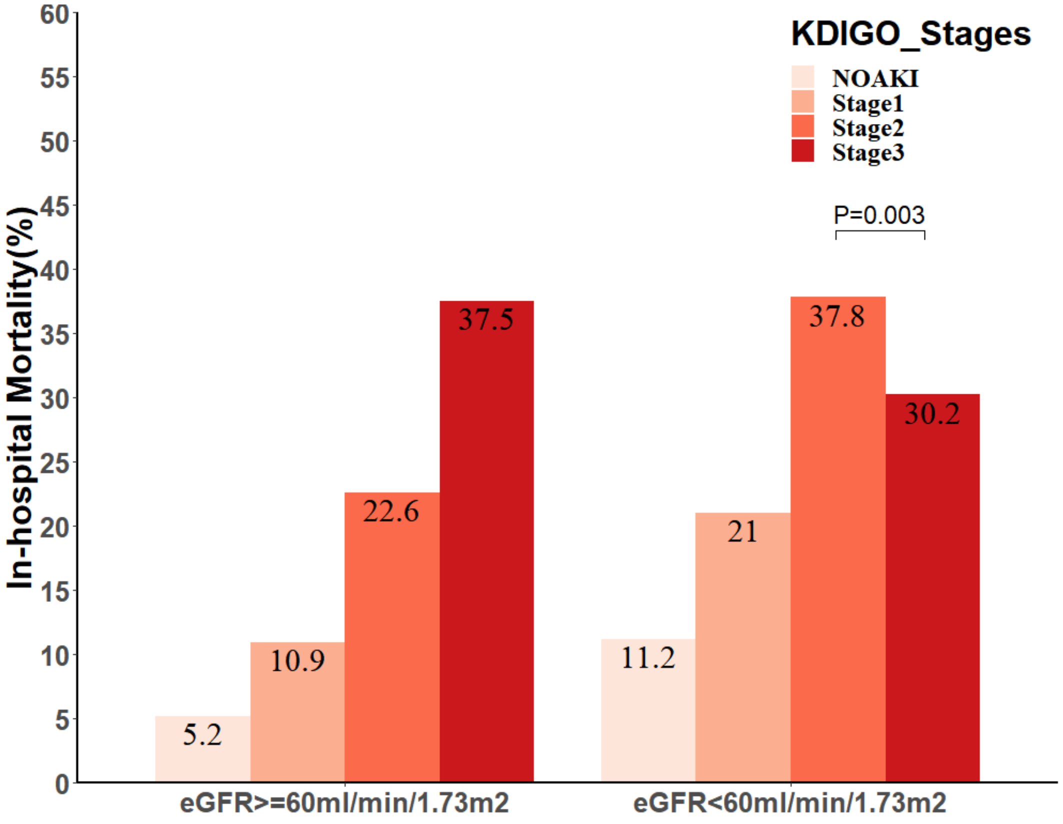

Figure S2. In-hospital mortality by KDIGO AKI severity stage in patients with and without PKD. AKI, acute kidney injury; PKD, preexisting kidney dysfunction (baseline eGFR <60 ml/min/1.73 m^2)

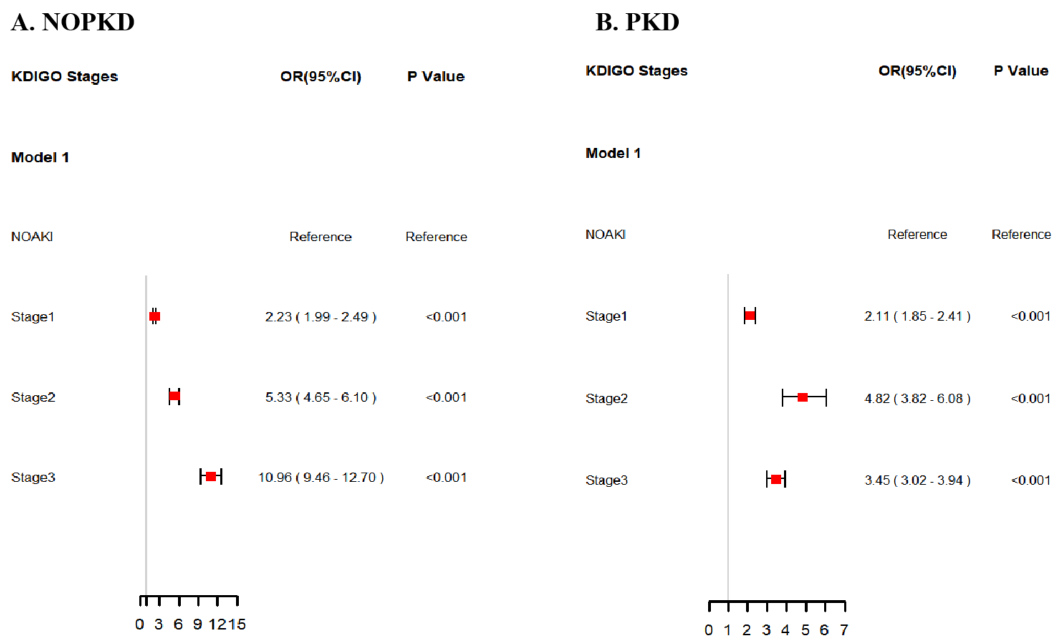

Figure S3. The Logistic regression analyses of in-hospital mortality by KDIGO AKI severity stage in patients with and without PKD. AKI, acute kidney injury; PKD, preexisting kidney dysfunction.

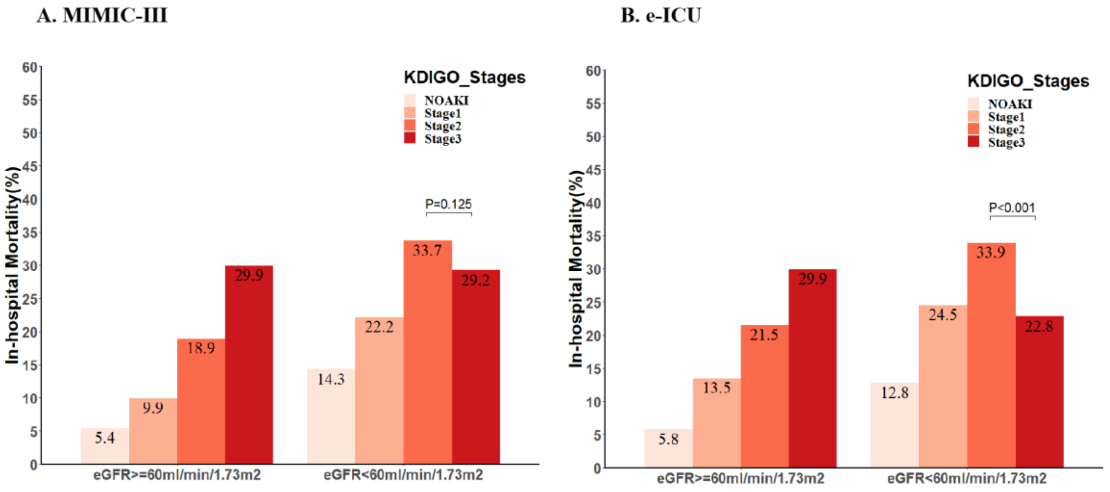

Figure S4. In-hospital mortality by KDIGO AKI severity stage in patients with and without PKD. AKI, acute kidney injury; PKD, preexisting kidney dysfunction.

A B

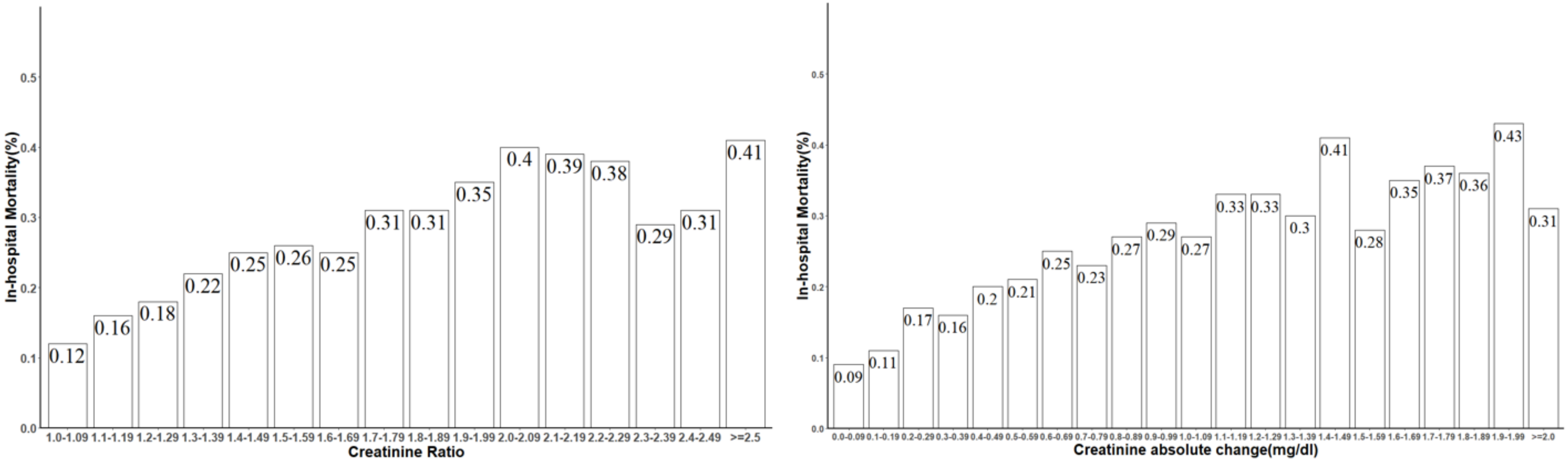

Figure S5. The in-hospital mortality varied with Creatinine Ratio and Creatinine absolute change.

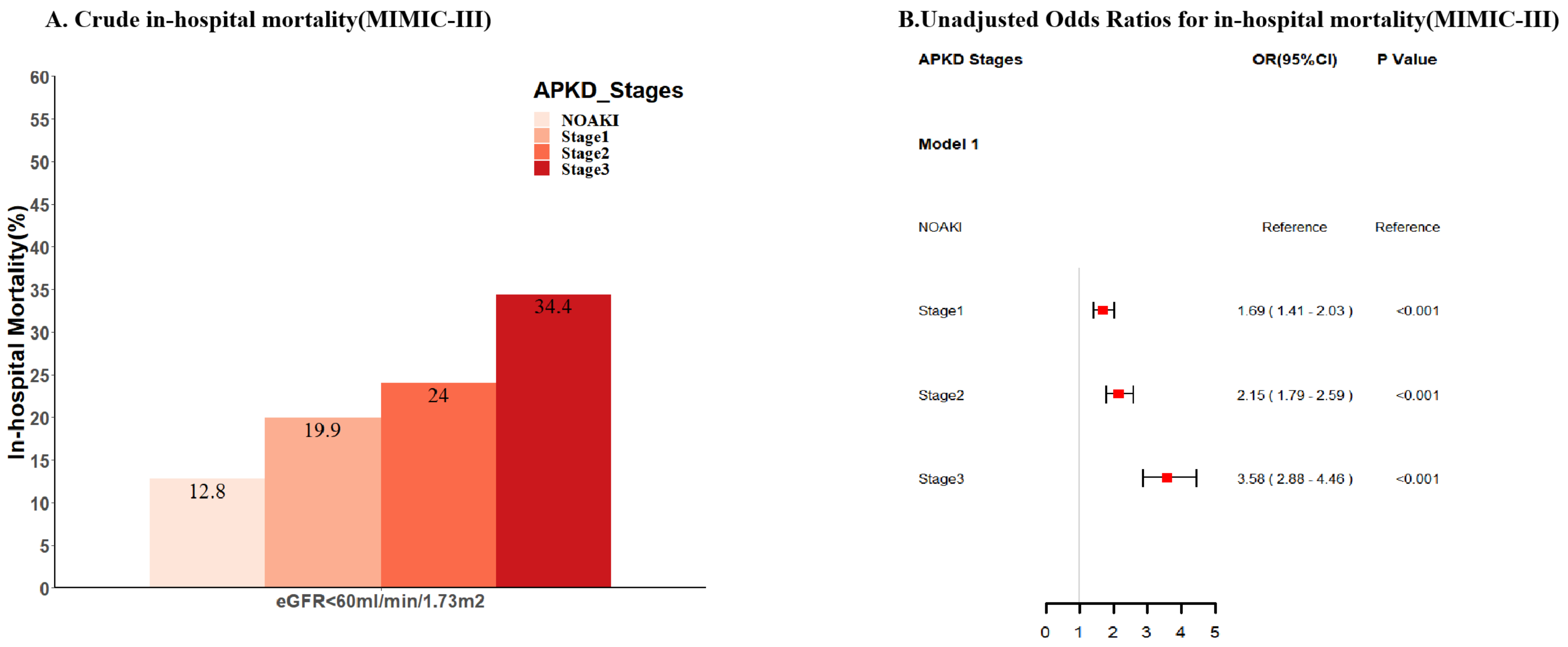

Figure S6. The crude and Logistic regression analyses of in-hospital mortality by APKD AKI severity stage in PKD patients in MIMIC-III cohort.

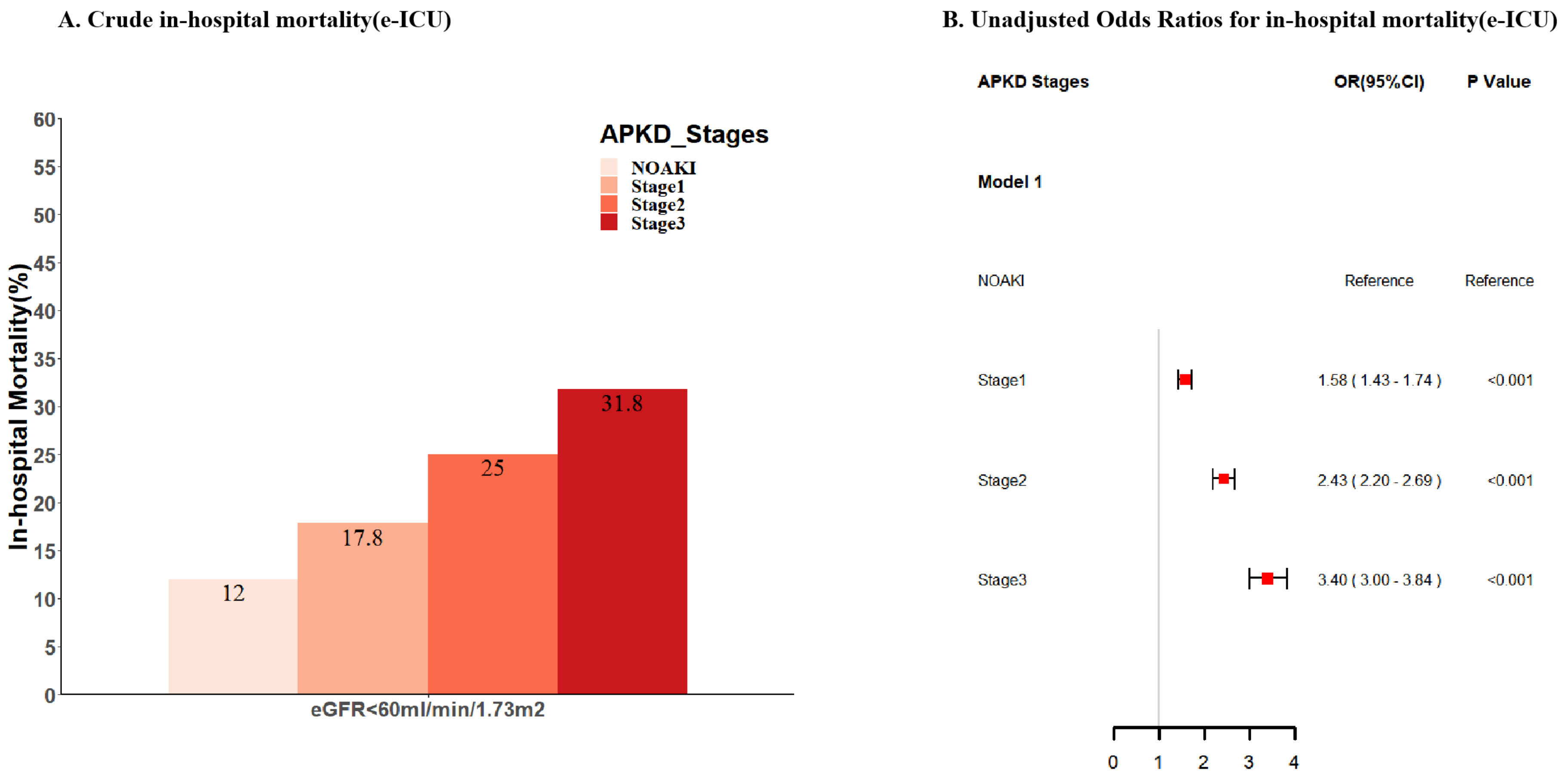

Figure S7. The crude and Logistic regression analyses of in-hospital mortality by APKD AKI severity stage in PKD patients in e-ICU cohort.

A. KDIGO stages

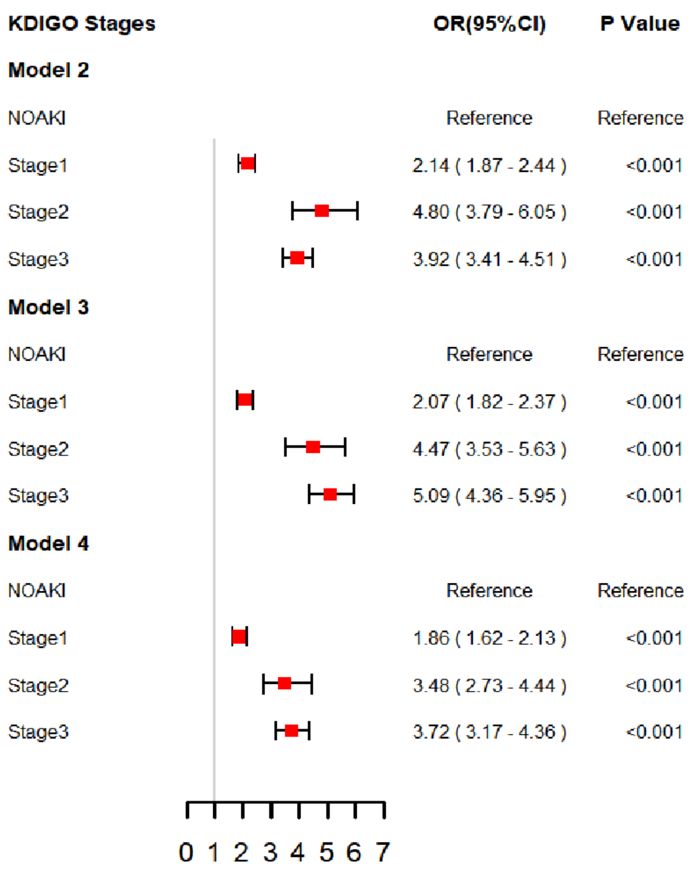

B. APKD stages

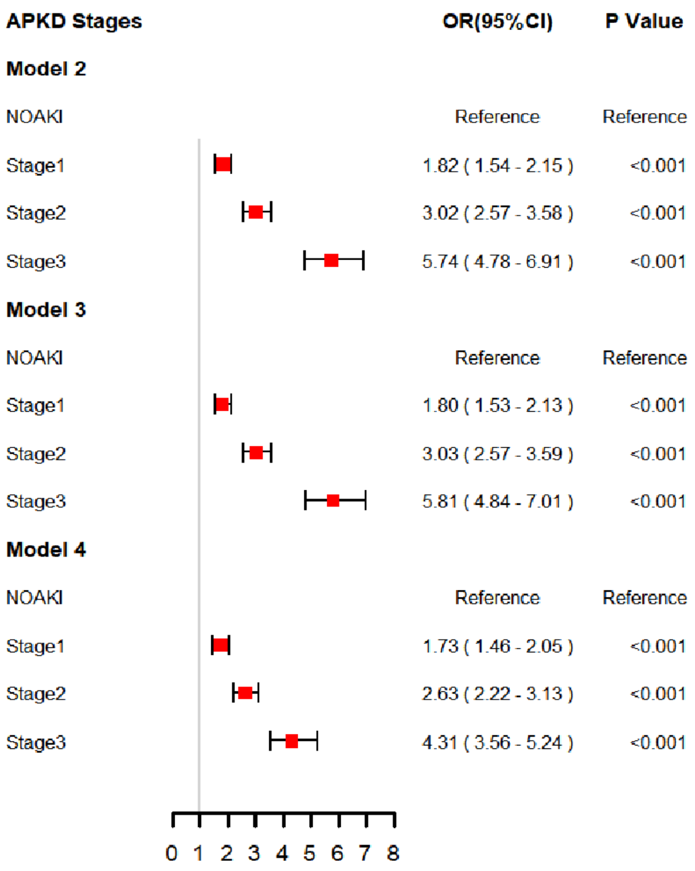

Figure S8. The Logistic regression analyses of in-hospital mortality by KDIGO and APKD AKI severity stage in patients with PKD.

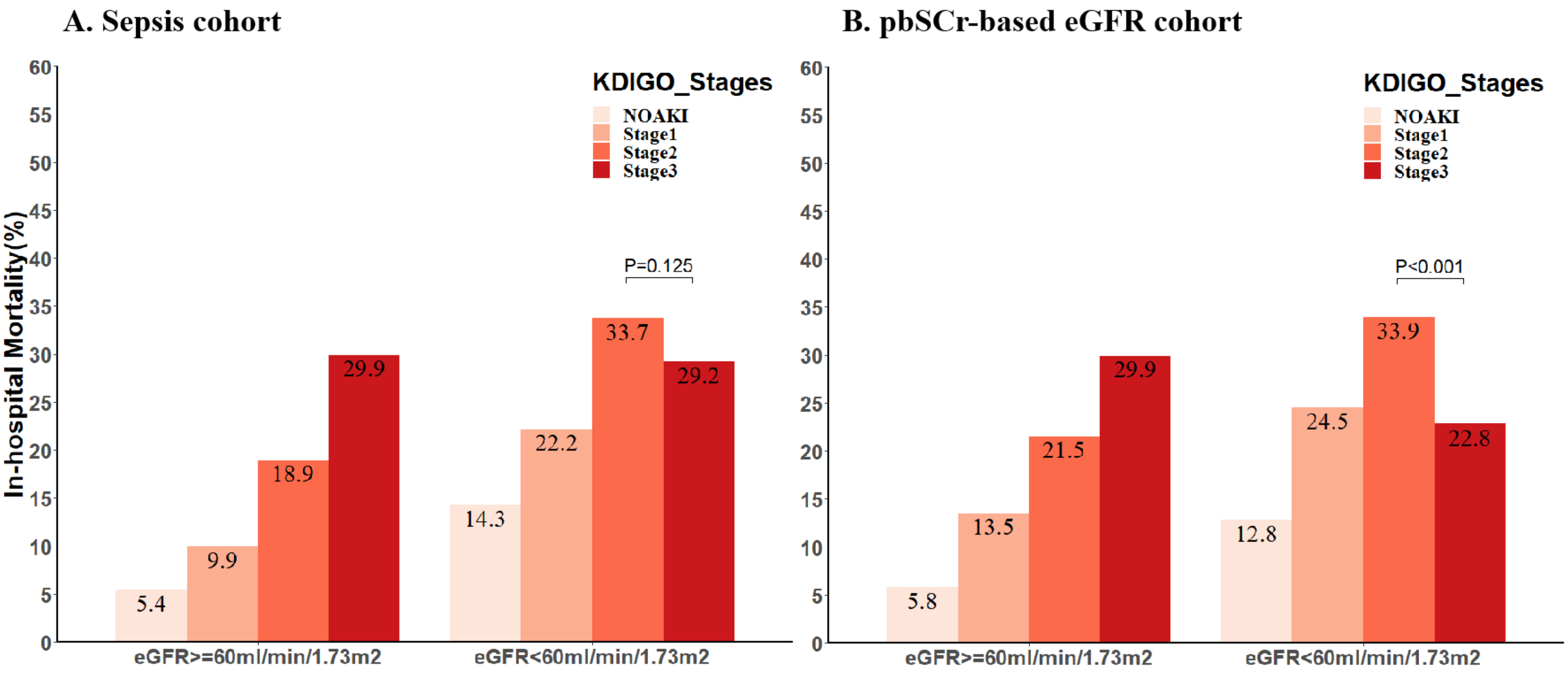

Figure S9. In-hospital mortality by KDIGO AKI severity stage in patients with and without PKD.

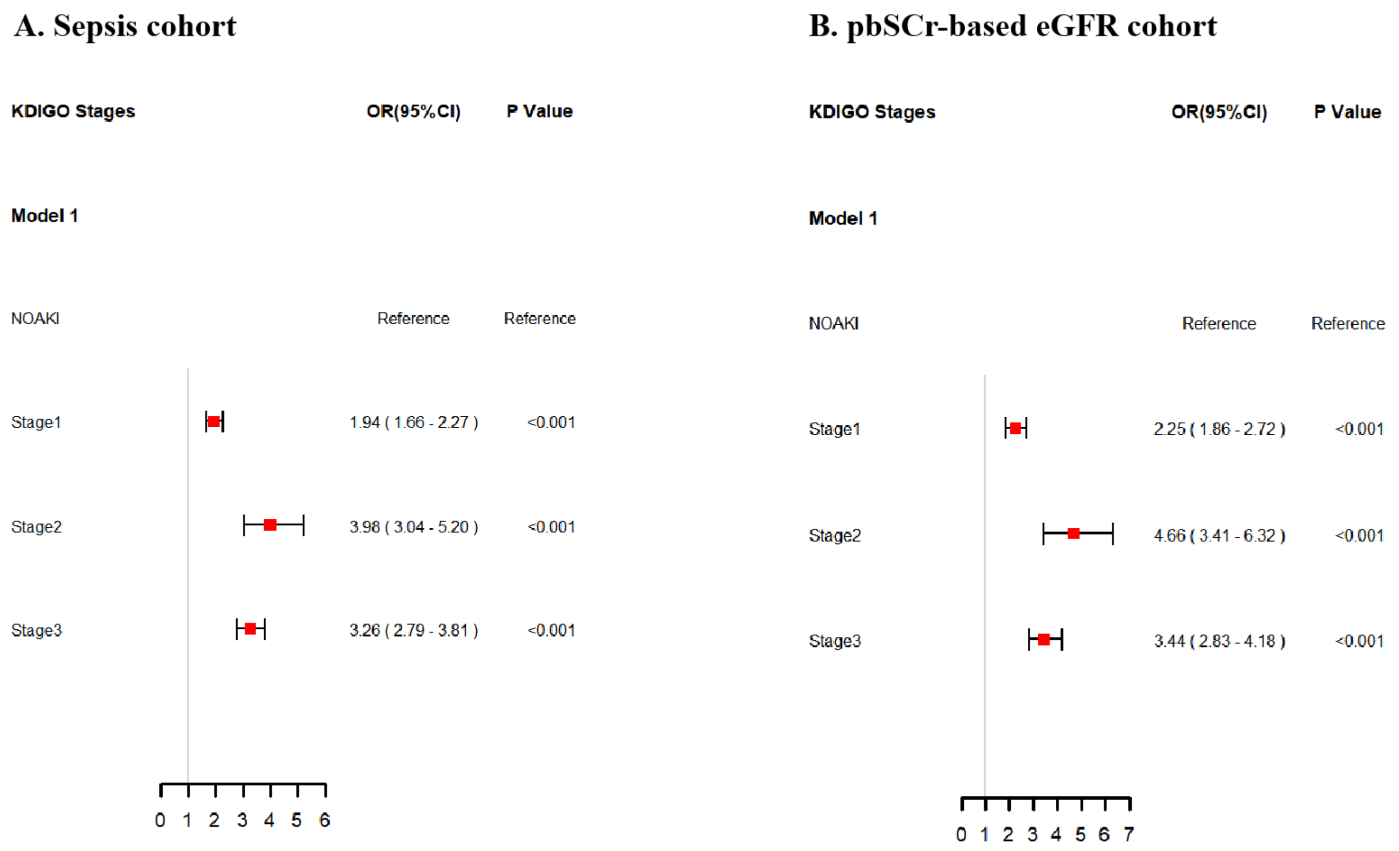

Figure S10. The Logistic regression analyses of in-hospital mortality by KDIGO AKI severity stage in patients with PKD.

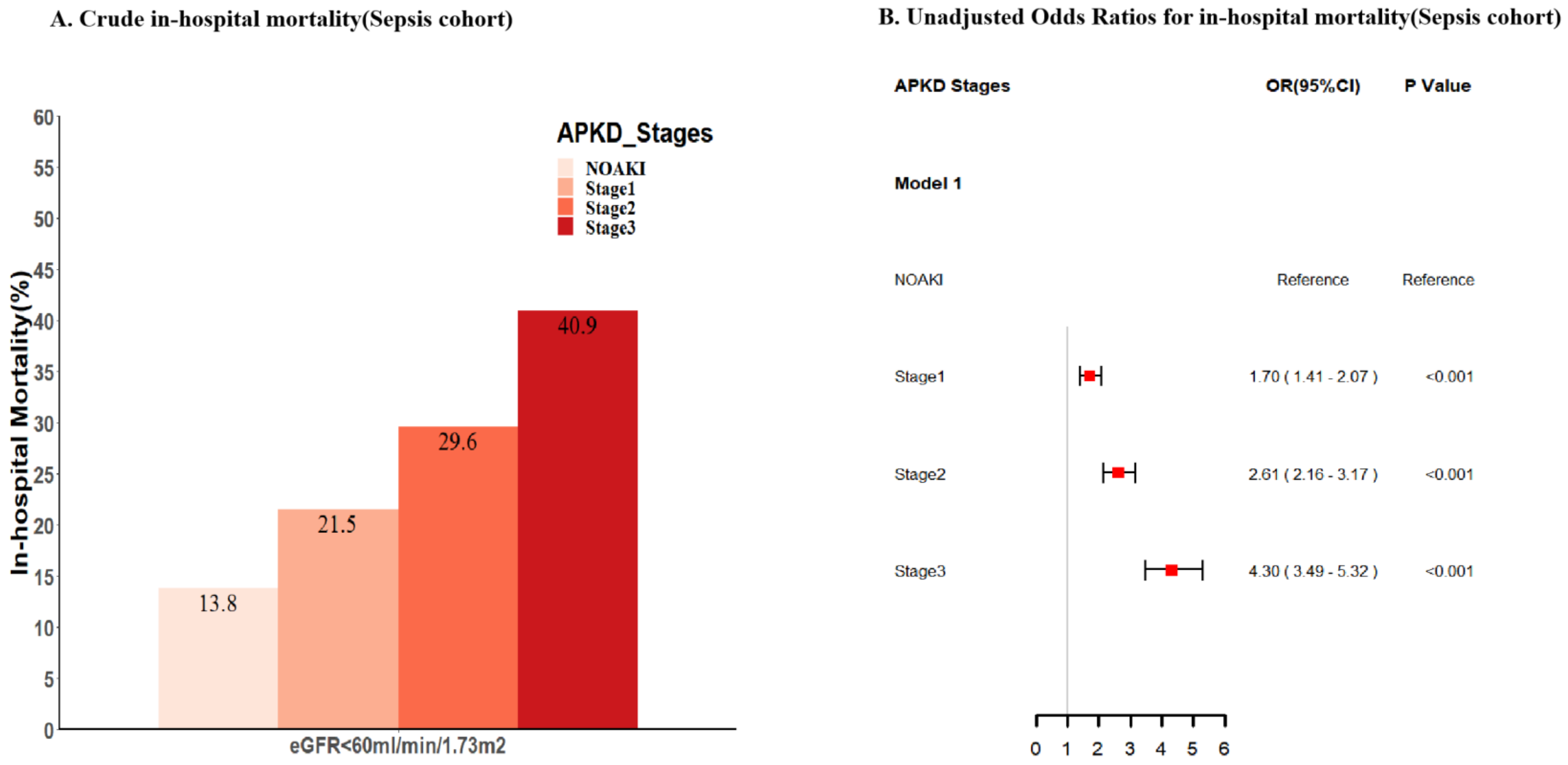

Figure S11. The crude and Logistic regression analyses of in-hospital mortality by APKD AKI severity stage in patients with PKD.

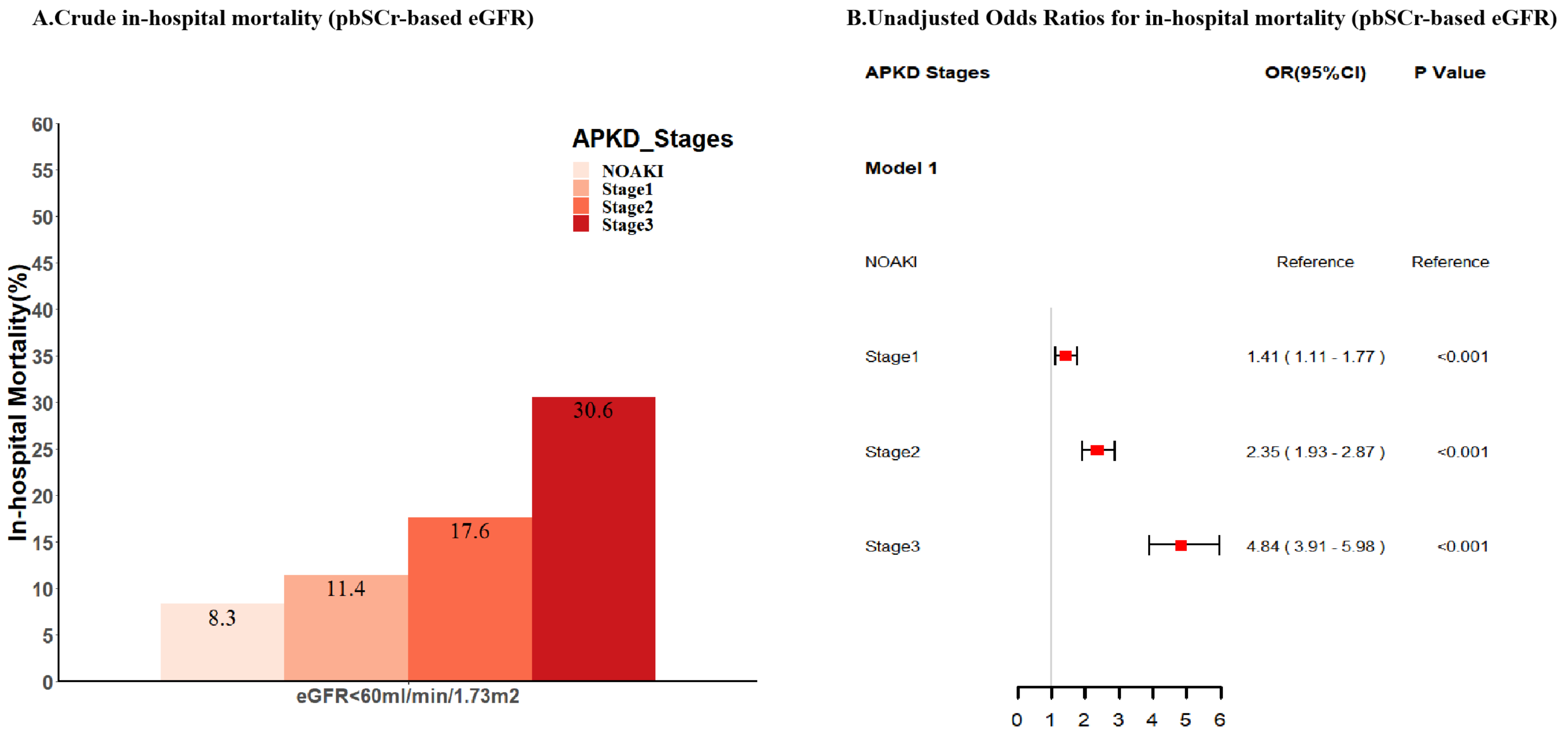

Figure S12. The crude and Logistic regression analyses of in-hospital mortality by APKD AKI severity stage in patients with PKD.
